# Mathematical modeling of radiotherapy: impact of model selection on estimating minimum radiation dose for tumor control

**DOI:** 10.3389/fonc.2023.1130966

**Published:** 2023-10-09

**Authors:** Achyudhan R. Kutuva, Jimmy J. Caudell, Kosj Yamoah, Heiko Enderling, Mohammad U. Zahid

**Affiliations:** ^1^ Department of Integrated Mathematical Oncology, H. Lee Moffitt Cancer Center & Research Institute, Tampa, FL, United States; ^2^ Department of Microbiology and Cell Science, University of Florida, Gainesville, FL, United States; ^3^ Department of Radiation Oncology, H. Lee Moffitt Cancer Center & Research Institute, Tampa, FL, United States

**Keywords:** radiotherapy, mathematical modeling, oncology, personalized oncology, model comparison

## Abstract

**Introduction:**

Radiation therapy (RT) is one of the most common anticancer therapies. Yet, current radiation oncology practice does not adapt RT dose for individual patients, despite wide interpatient variability in radiosensitivity and accompanying treatment response. We have previously shown that mechanistic mathematical modeling of tumor volume dynamics can simulate volumetric response to RT for individual patients and estimation personalized RT dose for optimal tumor volume reduction. However, understanding the implications of the choice of the underlying RT response model is critical when calculating personalized RT dose.

**Methods:**

In this study, we evaluate the mathematical implications and biological effects of 2 models of RT response on dose personalization: (1) cytotoxicity to cancer cells that lead to direct tumor volume reduction (DVR) and (2) radiation responses to the tumor microenvironment that lead to tumor carrying capacity reduction (CCR) and subsequent tumor shrinkage. Tumor growth was simulated as logistic growth with pre-treatment dynamics being described in the proliferation saturation index (PSI). The effect of RT was simulated according to each respective model for a standard schedule of fractionated RT with 2 Gy weekday fractions. Parameter sweeps were evaluated for the intrinsic tumor growth rate and the radiosensitivity parameter for both models to observe the qualitative impact of each model parameter. We then calculated the minimum RT dose required for locoregional tumor control (LRC) across all combinations of the full range of radiosensitvity and proliferation saturation values.

**Results:**

Both models estimate that patients with higher radiosensitivity will require a lower RT dose to achieve LRC. However, the two models make opposite estimates on the impact of PSI on the minimum RT dose for LRC: the DVR model estimates that tumors with higher PSI values will require a higher RT dose to achieve LRC, while the CCR model estimates that higher PSI values will require a lower RT dose to achieve LRC.

**Discussion:**

Ultimately, these results show the importance of understanding which model best describes tumor growth and treatment response in a particular setting, before using any such model to make estimates for personalized treatment recommendations.

## 1 Introduction

More than 50% of all cancer patients will receive radiation therapy (RT) during the course of their cancer treatment, either given with curative intent as a single agent, concurrently with systemic therapies, or (neo-)adjuvant to other therapeutic approaches, or in the palliative setting (1). Even modest improvements in treatment outcomes and quality of life for cancer patients undergoing RT would yield benefits for a large patient cohort. However, current radiation oncology practice does not personalize or adapt RT dose for individual patients, despite variance in individual patient radiosensitivity. Thus, many patients are potentially receiving either too much or too little RT dose. Recent efforts include more strategic integration of basic science approaches into radiobiology and radiation oncology to help better understand the mechanisms of radiation response dynamics and to help predict how to best personalize radiation to individual patients. Genomic signatures (2–5), imaging metrics (6–8), and burgeoning machine learning and artificial intelligence approaches are being retrospectively and prospectively evaluated as novel biomarkers for radiation response (9–11).

Simple mathematical approaches have a long history in radiobiology and radiation oncology. The linear-quadratic (LQ) model that describes the clonogenic survival of a cell population to increasing acute doses of radiation has been extensively used to identify cell-intrinsic radiosensitivities (12–14). Prominent developments of the LQ model include the concept of biologically effective dose (BED), tumor control probability (TCP), and normal tissue complication probability (NTCP) (15–19). Many conceptual studies have attempted to explain the biological underpinnings of linear-quadratic response dynamics. However, the non-linear tumor growth and treatment response dynamics require deployment of population dynamics models.

Mathematical oncology may hold the key to mechanistic understanding of the complex adaptive dynamic tumor system and its response to radiotherapy (20–24), with demonstrated feasibility of translation into prospective clinical trials. Using TCP and NTCP concepts combined with a logistic differential equation that describes the recovery of normal tissues from sublethal radiation-induced damage, Scott et al. pioneered the concept of temporally feathered radiation therapy (TFRT) that prioritizes and de-prioritizes organ-at-risk doses at different times during treatment. TFRT was subsequently shown to lead to increased doses to the radiation target, or reduced cumulative doses to organs at risk (25). Leder et al. combined experimental and differential equation models to identify novel radiation schedules to significantly improve radiation efficacy by taking advantage of the dynamic instability of radioresistance (26), which was recently demonstrated to be feasible and safe to administer to glioblastoma patients (27). Our group has introduced the concept of a patient specific ‘carrying capacity’ in a logistic growth model, called the proliferation saturation index (PSI) as a putative biomarker for radiosensitivity in head and neck cancer as well as non-small cell lung cancer (28–31) that is currently being evaluated as trigger for personalized radiation dose fractionation (NCT03656133).

One of the advantages of using mechanistic mathematical models to simulate radiation responses is that if an appropriate model is calibrated, validated, and predictive power demonstrated, then it may be used to simulate potential alternative treatments (32, 33). However, it is critical to examine the effects of the underlying models on these alternative treatment recommendations. While two models with different mechanstic mathematical formulations may be trained to fit longitudinal dynamics and predict individual responses equally well, they could have different implications for alternative radiation dose fractionations.

**Figure 1:**
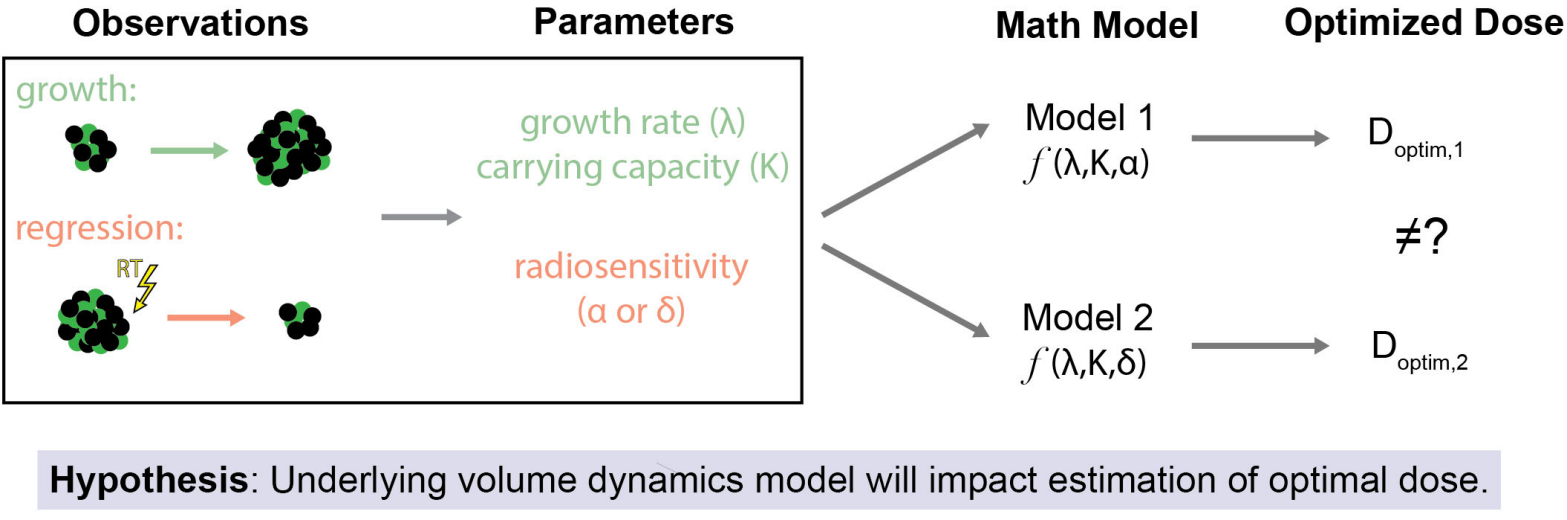
Study Overview and Hypothesis. The primary objective of this study is to assess how the selection of the underlying mathematical model of tumor volume dynamics affects estimates for optimal RT dose. This is premised on the idea that even when the same observations of tumor volume changes (both due to off-treatment growth and regression due to treatment effect) are used as inputs for differing models of response to RT there may be different estimates of the optimal RT dose.

Poleszczuk et al. previously analyzed that clinical predictions are strongly dependent on the specific growth law assumed, and that the applicable growth law should be known to be utilized in clinical practice (29). The objective of this paper is to examine the impact of the underlying tumor volume dynamics models on the estimated optimal RT dose (**Figure 1**). In a previous study (34), our group used a pre-specified model of tumor volume response to RT to estimate the minimum RT dose required for locoregional control of head and neck tumors. Herein, we compare two different mathematical models of response to RT: (1) cytotoxicity to cancer cells that lead to direct tumor volume reduction (DVR) and (2) radiation damage to the tumor microenvironment that lead to tumor carrying capacity reduction (CCR) and subsequent tumor shrinkage. Both of these models have been shown capable of fitting longitudinal tumor volume data from head and neck cancer patients (31, 35). The comparison is focused on evaluating the impacts of both of these models on RT dose personalization.

## 2 Methods

### 2.1 Tumor growth model

Tumor growth models are plentiful, ranging from incredibly simple exponential growth to highly different growth dynamics as the tumor volume changes, either relative to itself or its (static or dynamic) microenvironment (36). While classical investigations sought a universal tumor growth model (37–39), the search of tumor growth laws is still very much ongoing (40). The seminal study by Benzekry et al. demonstrated that the Gompertz growth model best captured pre-clinical *in vivo* breast and lung cancer growth dynamics but fell short of adequate forecasts beyond one subsequent measurement (41). More recently, Kather and his team provided the first such model comparison analysis in clinical data of 1,472 patients undergoing chemotherapy or cancer immunotherapy for solid tumors (42). Again, the Gompertz model provided the best balance between goodness-of-fit and number of parameters, but once more early treatment response was only moderately correlated with final treatment responses.

**Figure 2:**
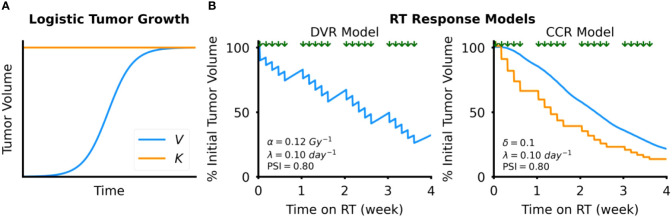
Tumor dynamics models. **(A)** Simulated example of tumor growth modeled as logistic growth. The blue curve shows tumor volume, *V*, over time and the orange line the tumor carrying capacity, *K* **(B)** Simulated examples of response to RT, which is modeled by either direct tumor volume reduction (DVR, left) or tumor carrying capacity reduction (CCR, right). Timing of RT fractions, simulated as a standard fractionated RT course with weekday fractions, is shown by the arrows above each plot.


We have previously shown that logistic growth dynamics provide excellent fits to clinical data of head and neck and non-small cell lung cancer during fractionated radiotherapy, and demonstrated predictive power of final tumor volumes with sufficient patient-specific data (30, 35). Logistic growth is described by the differential equation:


$$\frac{dV}{dt}=\lambda V\left(1-\frac{V}{K}\right)$$

where *V* is tumor volume (cc), *λ* is the intrinsic tumor growth rate (day^-1^), and *K* is the carrying capacity of the tumor (cc), which is the maximum size tumor that the local tissue can support (28). In logistic growth, the tumor volume grows initially exponentially but growth monotonically decelerates as the volume approaches the defined carrying capacity, visually indicated by the horizontal asymptote (**Figure 2A**). In describing the distinct types of growth dynamics that this model can capture, our group has previously defined the proliferation saturation index (PSI) a measure of the effective tumor growth rate in the absence of RT (28). PSI is defined by the expression:

$$PSI\;\equiv \frac{V_{0}}{K_{0}}$$

where $V_{0}$ and *K _0_* are the initial tumor volume and tumor carrying capacity before treatment, respectively. *K_0_* can be prospectively calculated using the following expression:


$$K_{0}=\frac{V_{0}V_{Dx}\left(e^{\lambda \text{Δ}t}-1\right)}{V_{Dx}e^{\lambda \text{Δ}t}-1}$$

where $V_{0}$ is the current tumor volume before radiation (usually obtained at radiation simulation), $V_{Dx}$ is the tumor volume measure at diagnosis, and *Δt* is the time between the two volume measurements (usually a few weeks).^5^ PSI is defined between 0 and 1. As PSI approaches 0 tumor growth approaches exponential growth, which indicates a tumor microenvironment capable of sustaining a much larger tumor than what currently exists. In contrast, as PSI approaches 1, tumor growth approaches its carrying capacity, which corresponds to high tumor proliferation saturation in the constraints of the tumor microenvironment limiting further proliferation of the tumor.

### 2.2 Modeling response to radiotherapy

We simulate the effect of RT with 2 different models (1): Direct Tumor Volume Reduction (DVR) and (2) Tumor Carrying Capacity Reduction (CCR).

#### 2.2.1 Direct tumor volume reduction model

In the DVR model (**Figure 2B**), we simulate the effect of an RT fraction as an instantaneous reduction in proliferating tumor volume due to cancer cell death:


$$V_{+}=\;V_{-}\left(1-\gamma \;\left(1-\frac{V_{-}}{K}\right)\right)$$

where *V_+_* is the tumor volume after the RT fraction; *V_-_* is the tumor volume before the RT fraction; γ is the cancer cell death rate; and *K* is the tumor carrying capacity. The parameter γ is derived from the linear-quadratic model (12, 43):


$$\gamma =1-e^{-\left(\alpha d\;+\;\beta d^{2}\right)}$$

where *d* [Gy] is the RT dose per fraction, and α [Gy^-1^] and β [Gy^-2^] are the LQ radiation sensitivity parameters, respectively. In this study, we set the ratio $\frac{\alpha }{\beta }$ = 10 Gy, as seen in many cancer types that are treated with fractionated RT, including head and neck cancer (44). Of note, here we model the effect of radiation on tumor volume and not individual cells in a clonogenic assay. Therefore, the absolute value of α may not be directly comparable to the preclinical radiobiology literature.

#### 2.2.2 Tumor carrying capacity reduction model

In the CCR model (**Figure 2B**), we model the effect of RT as an instantaneous reduction in the tumor carrying capacity:


$$K_{+}=K_{-}\;\left(1-\delta \right)$$

where K_+_ is the tumor carrying capacity after each RT fraction; K_–_ is the tumor carrying capacity before the RT fraction; and δ is the proportion that the carrying capacity is reduced with each RT fraction, ranging from 0 to 1. Modeling the effect of RT as a reduction in the tumor carrying capacity is motivated by observations of how RT alters components of the tumor microenvironment, such tumor vasculature (45) or the release of tumor-specific antigens and damage-associated molecular patterns (DAMPs) that stimulate antitumor immunity (46), that may reduce the tumor carrying capacity. As of yet, the actual dose dependency of radiation-induced carrying capacity reduction is unknown. Thus, we limit this study to the effect of the total dose given in 2 Gy fractions, without consideration of alternative dose fractionations.

### 2.3 Simulating tumor volume dynamics during RT

All simulations were done using custom scripts developed in Java and subsequent analyses were done in Python. The code for both is available at the following Github repository: https://github.com/akutuva21/SPARK-Project. All simulations of RT were performed using a common schedule for fractionated RT, where *d* = 2 Gy fractions are delivered daily Monday-Friday with no RT delivered on Saturday and Sunday. All simulations of RT were performed using schedules and doses routinely used in treating head and neck cancer patients. Between-fraction tumor volume changes were simulated with the logistic growth model using a 1-hour time resolution.

### 2.4 Parameter sweep analysis

To understand the impact of the model parameters on tumor volume dynamics, we conducted parameter sweeps of both the radiosensitivity parameters (α for the DVR model and δ for the CCR model) and the intrinsic tumor growth rate, λ. For the radiosensitivity parameter sweeps, we set λ = 0.1 day^-1^ and PSI = 0.9 for all simulations. These parameters are arbitrarily chosen to investigate qualitative response dynamics. Dynamics for different growth rate and PSI parameters are comparable and intuitively derivable from the below results. For the DVR model, we tested α ∈ (0, 0.20) Gy^-1^, with a step size of 0.01 Gy^-1^. For the CCR model, we tested δ ∈ (0, 0.20), with a step of size of 0.01.

For the intrinsic growth rate (λ) sweeps, we tested λ ∈ (0, 0.10) day^-1^ for both models. For the other parameters, we set a PSI = 0.7 for both models for rich model dynamics, and α = 0.1 Gy^-1^ for the DVR model and δ = 0.1 for the CCR model. These sweeps were done using simulations of standard 6 weeks of RT with standard weekday fractionation. Parameter ranges were informed by previous studies fitting these models to longitudinal tumor volume data from head and neck cancer patients that received fractionated RT (31, 34, 35). However, herein we focus on qualitatively demonstrating response dynamics without emphasis on actual values for a specific cancer type.

### 2.5 Estimating minimum RT dose for locoregional tumor control

In head and neck cancer, mid-treatment volumetric responses correlate with outcome (31, 47). Patients with greater than 32.2% tumor volume reduction after 4 weeks of RT were 100% locoregionally controlled (LRC) at a mean follow-up time of 20 months. We have previously used this tumor volume reduction threshold to estimate patient-specific RT doses to achieve locoregional control (LRC) using the CCR model (34). While it is conceivable that a greater tumor volume reduction would not jeopardize tumor control, higher RT doses are correlated with higher normal tissue complication probability (NTCP).

Here, we will use the 32.2% tumor volume reduction threshold to estimate the minimum cumulative RT dose required to achieve locoregional control (LRC) in both DVR and CCR models. We simulate RT up to 8 weeks (allowing consideration of modest dose escalation) using the same fractionation schedule and dose/fraction described above and then finding the minimum cumulative dose (*D*_min_) where the tumor volume shrinks below the volume reduction threshold. These simulations were done over the following parameter ranges: PSI ∈ (0.6,1.0), α ∈ (0.06, 0.14) Gy^-1^ for the DVR model, and δ ∈ (0.01, 0.09) for the CCR model.

## 3 Results

### 3.1 Parameter sweep analysis

**Figure 3:**
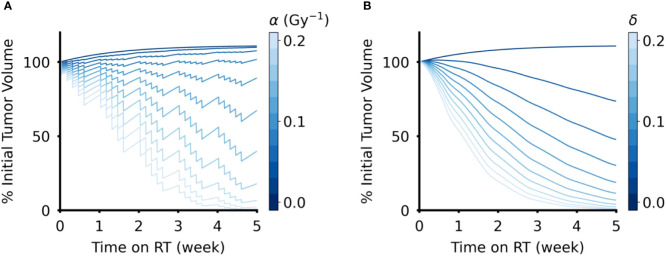
Effect of radiation sensitivity parameter in DVR and CCR models. **(A)** Tumor volume trajectories simulated using the DVR model with values of α ∈ (0,0.2) Gy^-1^, where larger α values lead to greater decrease in tumor volume. **(B)** Tumor volume trajectories simulated using the CCR model with values of δ ∈ (0,0.2), where larger δ values lead to greater decrease in tumor volume. For both models, λ = 0.1 day^-1^ and PSI = 0.9, and the value of the respective radiation sensitivity parameters are indicated by the color bar. All simulations have an arbitrary initial tumor volume with a fractionated RT regimen, where treatment is applied every weekday for a total of five weeks of treatment.

**Figure 4:**
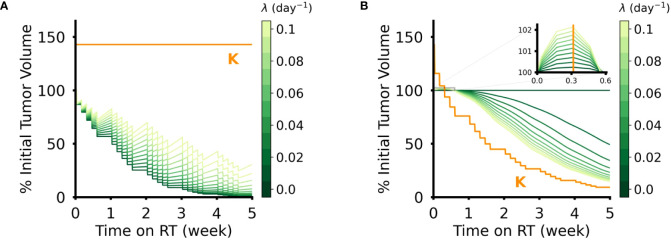
Effect of intrinsic tumor growth rate, λ, in DVR and CCR models. **(A)** Tumor volume trajectories simulated using the DVR model with α = 0.1 Gy^-1^ and λ ∈ (0,0.1) day^-1^. Lower λ values lead to greater net reduction in tumor volume at the end of the treatment course. **(B)** Tumor volume trajectories simulated using the CCR model with δ = 0.1 and λ ∈ (0,0.1) day^-1^. Higher λ values lead to greater net reduction in tumor volume at the end of the treatment course. The inset shows the initial phase of simulated RT, where the tumor volume remains above the carrying capacity and lower λ still results in lower tumor volumes. For all simulations PSI = 0.7, and the values of λ are indicated by the color bar. All simulations have an arbitrary initial tumor volume with a fractionated RT regimen, where treatment is applied every weekday for a total of five weeks of treatment.

Intuitively, when the radiosensitivity parameters (α for the DVR model, δ for the CCR model) increase, the reduction in tumor volume increased for both models of RT response (**Figure 3**). However, the effect of the intrinsic growth rate, λ, on tumor volume reduction were opposite in the two models. In the DVR model, as λ decreases the net tumor volume reduction increases (**Figure 4A**). This is because with lower λ values there is less repopulation between RT fractions. In the CCR model, however, higher λ values result in higher net tumor volume reduction (**Figure 4B**). This counterintuitive result comes from the fact that in the CCR model tumor volume only decreases when *V* > *K*, which makes $\frac{dV}{dt}$ < 0 and results in λ becoming the rate at which the tumor volume approaches the current carrying capacity from above. Of interest, this contrasts with the response dynamics during the first week of RT (**Figure 3B**, inset). During early radiation fractions, the carrying capacity remains greater than the tumor volume, which results in $\frac{dV}{dt}$ > 0 and continued tumor growth, albeit slower with each fraction as V approaches K. Thus, initially, higher λ values yield higher transient tumor volumes, followed by steeper volume reduction.

### 3.2 Minimum cumulative dose estimation

**Figure 5:**
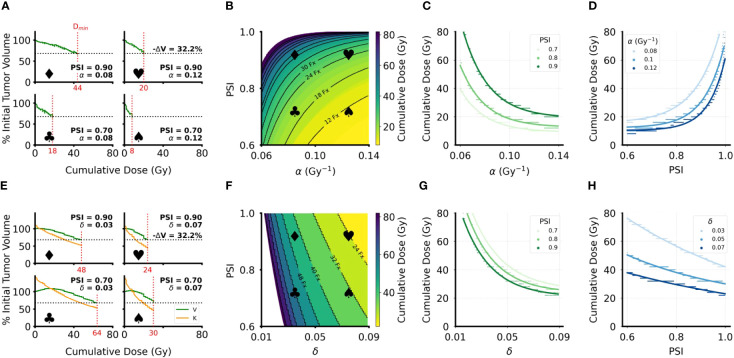
Minimum cumulative dose (D_min_) for LRC estimates for DVR and CCR models. **(A, E)** Sample volume trajectories for representative parameter pairs across the parameter range, where the bold symbols indicate the location on the heatmap in **(B, F)**. Green curves are the tumor volume plotted as function of cumulative dose, which increases linearly with treatment time; horizontal dashed line indicates the 32.2% volume reduction cutoff used to calculate D_min_; vertical red dashed line indicates the calculated D_min_ with the specific value of D_min_ indicated on the x-axis. Patient-specific parameters for each simulation are indicated on the corresponding plots. For all simulations, λ = 0.07 day^-1^. **(B, F)** Heatmaps of D_min_ over the clinically relevant range for the radiosensitivity parameter (α or δ) and PSI. All simulations have an arbitrary initial tumor volume with a fractionated RT regimen, where treatment is applied every weekday for a total of five weeks of treatment. Black curves indicate “iso-dose” levels with the number of RT fractions required for the indicated dose. White areas indicate parameter regions where sufficient volume reduction was not achieved in the 8 weeks of simulated RT. **(C, G)** Plots of the radiosensitivity parameters (α or δ) against D_min_ for PSI = 0.7, 0.8, 0.9. Colored dots are data points sampled from the heatmaps in **(B**, **F)**; the corresponding solid lines are exponential fits to the data (fitted coefficients in SI). **(D, H)** Plots of PSI against D_min_ for 3 different values of the radiosensitivity parameters. Colored dots are data points sampled from the heatmaps in **(B, F)**; the corresponding solid lines are exponential fits to the data (fitted coefficients in **SI Tables 1**–**4**).

In the DVR model, higher radiosensitivity (α) leads to lower estimated *D*_min_ for LRC, while higher PSI values lead to higher estimated *D*_min_ (**Figures 5A, B**). Similarly, in the CCR model higher radiosensitivity (δ) leads to lower estimated *D*_min_ for LRC. However, increasing PSI in the CCR model leads to lower estimated *D*_min_ for LRC (**Figures 5E, F**). This is, again, due to tumor reduction being achieved only when the carrying capacity drops below the current tumor volume, and consequently $\frac{dV}{dt}$ < 0. The closer the tumor volume is to its carrying capacity (i.e., higher PSI), the faster radiation can reduce the carrying capacity below the current value. The implications of these analyses can be more clearly seen by looking at *D*_min_ for LRC as a function of PSI and the radiosensitivity parameters (**Figures 5C, D, G, H**). *D*_min_(*PSI*), *D*_min_(*α*), and *D*_min_(*δ*) were fit to exponential functions with the form $\;D_{min}=a\cdot e^{bx}+c$ , where *x* is PSI, α, or δ depending on the respective context (fitted coefficient values in **SI Tables 1**–**4**). In the DVR model, higher PSI always yields a higher estimate for *D*_min_ regardless of the value of α (**Figure 5C**). The opposite is true in the CCR model, where higher PSI always yields a lower estimate for *D*_min_, regardless of the value of δ (**Figure 5G**). Additionally, for both the DVR and CCR model, higher values of the radiosensitivity parameter yield lower estimates for *D*_min_ (**Figures 5D, H**). Overall, in the DVR model the highest estimated *D*_min_ values are found at high PSI values and low α values, while in the CCR model the highest estimated *D*_min_ values are found at low δ values and low PSI values.

## 4 Discussion

In this study we have shown that different mathematical models of RT response yield different clinical implications. Thus, the choice of RT response model is critical when estimating RT doses that best shrink tumor volumes based on intrinsic model parameters. Although the two models studied herein both estimate that patients with higher radiosensitivity will require a lower RT dose to achieve LRC, they make opposite estimates on the impact of pre-treatment tumor growth dynamics biomarker, PSI. Fast-growing tumors have lower PSI values and slow-growing tumors have higher PSI values. The DVR model, which assumes that the effect of RT comes from the direct radiation-induced death of tumor cells, estimates that tumors with higher PSI values, i.e. lower pre-treatment proliferation, will require a higher RT dose to achieve LRC. However, the CCR model, which assumes that the effect of RT comes from a reduction in the tumor carrying capacity of the local tissue, estimates that higher PSI values will require a lower RT dose to achieve LRC. It is therefore of utmost importance to know which mathematical model best describes the radiobiology that underlies the observable radiation response dynamics. It is encouraging, however, that both radiosensitivity and PSI could be measured or estimated in the clinic: PSI can be calculated by using two temporally separated tumor volume measurements before the start of treatment (28) and radiosensitivity via genomic measures such as the radiosensitivity index (48). Estimates of radiosensitivity may increase in accuracy by using serial measurements of the tumor volume during RT to dynamically update estimates of tumor radiation response (35).

The opposing estimates of the effect of PSI on the required minimum RT dose for LRC stem from the fact that tumor cell death is modulated by PSI in the DVR model as the model assumes that only proliferative cells are killed by RT. This means that as PSI increases, higher and higher doses will be required to achieve the same reduction in tumor volume. On the other hand, in the CCR model tumor volume reduction only occurs once the carrying capacity is less than the tumor volume. This means that tumors with higher PSI values require less RT dose for the carrying capacity to drop below the tumor volume, as the initial values for *V* and *K* are already relatively close to each other. This result suggests that by determining which model more accurately describes on-treatment tumor volume dynamics in a particular scenario it may be possible to determine which effect of RT is more dominant.

Herein, we focused on two particular models of radiation response mechanisms in the logistic growth model and PSI framework that have been previously presented – and studied which of the two mechanisms has the predominant effect tumor volume dynamics (28, 35). While it is conceivable that both mechanisms – direct cancer cell kill and modulation of the tumor microenvironment via carrying capacity reduction – contribute to the clinically observed tumor responses, the current framework unable to combine both models without significant adjustments to the underlying mathematics. Combining both models (SI Methods) leads to scenarios where V/K > 1 resulting in numerical artifacts of unrealistic spikes and large oscillations in tumor volume (**SI Figure 1**) for the majority of tested model parameters (**SI Figure 2**). It may be possible to prevent this issue by simulating DVR and CCR on different timescales, but the required mathematics are beyond the scope of this study and left for future analysis.

Furthermore, we have constrained the models to only simulate changes in tumor volume immediately before and during an RT treatment course. There are documented delayed and cumulative effects of RT that manifest in the months following radiation (49–51). The discussed models, however, are specifically trained to simulate on treatment tumor response dynamics. For tumor decay following radiation, for example due to activated immune responses or clearance of necrotic debris, more complex models will be required that do not contribute to the implications of the herein discussed results. Additionally, the chosen models require measurable tumor volume. Any dynamics below the limit of detection where stochastic effects may dominate need to be simulated with different mathematical approaches (52, 53).

Ultimately, it is critical to understand if a model appropriately describes tumor growth and treatment response for specific cancer subtypes, or individual patients, before using any such model to make estimates for personalized treatment recommendations. It may eventually be possible to select appropriate patient-specific or tumor site-specific models, but this will require further study. One route for studying which models of response to RT are best fit for different contexts will be increased acquisition of both direct and indirect measurements of tumor burden during the course of RT. This may be enabled by emerging techniques such as RT with MRI-guided linear accelerators (MR-LinAc) (54, 55) and liquid biopsies to measure circulating tumor DNA (ctDNA) and cell-free DNA (cfDNA) (56–58). In the absence of sufficient evidence for cancer-specific or patient-specific model selection, however, the more prudent approach may be to consider model ensembles for making prediction or treatment recommendations. Ensemble modeling is commonly utilized in weather forecasting, transport modeling, ecology, or financial forecasting to account for model biases, measurement uncertainties, and forecast uncertainty (59–63). In the context of modeling tumor response to RT, if using an ensemble of models, one might only recommend a change from standard treatment or dosing when a sufficient number of models in the ensemble agree on the direction or magnitude of the estimated treatment personalization.

## Data availability statement

The raw data supporting the conclusions of this article will be made available by the authors, without undue reservation.

## Author contributions

AK, MZ, and HE contributed to the conceptualization and design of the study. AK performed all modelling and analyses. JC and KY provided clinical insight and feedback. HE and KY provided administrative and material support. AK, MZ, and HE co-wrote the first draft of the manuscript. MZ supervised the study. All authors contributed to the article and approved the submitted version.

## References

[1] Delaney G Jacob S Featherstone C Barton M. The role of radiotherapy in cancer treatment: estimating optimal utilization from a review of evidence-based clinical guidelines. Cancer: Interdiscip Int J Am Cancer Soc (2005) 104(6):1129–37. doi: 10.1002/cncr.21324 16080176

[2] Torres-Roca JF. A molecular assay of tumor radiosensitivity: a roadmap towards biology-based personalized radiation therapy. Per Med (2012) 9(5):547–57. doi: 10.2217/pme.12.55 PMC3480204 23105945

[3] Eschrich S Zhang H Zhao H Boulware D Lee JH Bloom G. Systems biology modeling of the radiation sensitivity network: A biomarker discovery platform. Int J Radiat Oncol Biol Phys (2009) 75(2):497–505. doi: 10.1016/j.ijrobp.2009.05.056 19735874 PMC2762403

[4] Eschrich SA Fulp WJ Pawitan Y Foekens JA Smid M Martens JWM. Validation of a radiosensitivity molecular signature in breast cancer. Clin Cancer Res (2012) 18(18):5134–43. doi: 10.1158/1078-0432.CCR-12-0891 PMC3993974 22832933

[5] Mellon E Yue B Strom TS Torres-Roca JF Fulp WJ Scott JG. A genome-based model for adjusting radiotherapy dose (GARD): a retrospective, cohort-based study. Lancet Oncol (2016) 18(2):202–11. doi: 10.1016/S1470-2045(16)30648-9 PMC7771305 27993569

[6] Fang M Kan Y Dong D Yu T Zhao N Jiang W. Multi-habitat based radiomics for the prediction of treatment response to concurrent chemotherapy and radiation therapy in locally advanced cervical cancer. Front Oncol (2020) 10:563. doi: 10.3389/fonc.2020.00563 32432035 PMC7214615

[7] Carles M Fechter T Radicioni G Schimek-Jasch T Adebahr S Zamboglou C. FDG-PET radiomics for response monitoring in non-small-cell lung cancer treated with radiation therapy. Cancers (2021) 13:814. doi: 10.3390/cancers13040814 33672052 PMC7919471

[8] Cozzi L Comito T Fogliata A Franzese C Franceschini D Bonifacio C. Computed tomography based radiomic signature as predictive of survival and local control after stereotactic body radiation therapy in pancreatic carcinoma. PloS One (2019) 14(1):e0210758. doi: 10.1371/journal.pone.0210758 30657785 PMC6338357

[9] el Naqa I Murphy MJ. What is machine learning? Machine learning in radiation. Oncology (2015), 3–11. doi: 10.1007/978-3-319-18305-3_1

[10] Tseng HH Luo Y ten Haken RK el Naqa I. The role of machine learning in knowledge-based response-adapted radiotherapy. Front Oncol (2018) 8:266. doi: 10.3389/fonc.2018.00266 30101124 PMC6072876

[11] Cui S Hope A Dilling TJ Dawson LA ten Haken R el Naqa I. Artificial intelligence for outcome modeling in radiotherapy. Semin Radiat Oncol (2022) 32(4):351–64. doi: 10.1016/j.semradonc.2022.06.005 36202438

[12] Fowler JF. The linear-quadratic formula and progress in fractionated radiotherapy. Br J Radiol (1989) 62:679–94. doi: 10.1259/0007-1285-62-740-679 2670032

[13] Dale RG. The application of the linear-quadratic dose-effect equation to fractionated and protracted radiotherapy. Br J Radiol (1985) 58(690):515–28. doi: 10.1259/0007-1285-58-690-515 4063711

[14] Brenner DJ. The linear-quadratic model is an appropriate methodology for determining isoeffective doses at large doses per fraction. Semin Radiat Oncol (2008) 18(4):234–9. doi: 10.1016/j.semradonc.2008.04.004 PMC2750078 18725109

[15] Dahlman EL Watanabe Y. Evaluating the biologically effective dose (BED) concept using a dynamic tumor simulation model. Med Phys (2020) 47(8):3710–20. doi: 10.1002/mp.14228 32385934

[16] Fowler JF. 21 Years of biologically effective dose. Br J Radiology. (2010) 83(991):554–68. doi: 10.1259/bjr/31372149 PMC3473681 20603408

[17] Palma G Monti S Conson M Pacelli R Cella L. Normal tissue complication probability (NTCP) models for modern radiation therapy. Semin Oncol (2019) 46(3):210–8. doi: 10.1053/j.seminoncol.2019.07.006 31506196

[18] Yaes RJ. Some implications of the linear quadratic model for tumor control probability. Int J Radiat Oncol Biol Phys (1988) 14(1):147–57. doi: 10.1016/0360-3016(88)90062-4 3335449

[19] Wennberg BM Baumann P Gagliardi G Nyman J Drugge N Hoyer M. NTCP modelling of lung toxicity after SBRT comparing the universal survival curve and the linear quadratic model for fractionation correction. Acta Oncol (Madr) (2011) 50(4):518–27. doi: 10.3109/0284186X.2010.543695 21198416

[20] Anderson ARA Quaranta V. Integrative mathematical oncology. Nat Rev Cancer (2008) 8(3):227–34. doi: 10.1038/nrc2329 18273038

[21] Altrock PM Liu LL Michor F. The mathematics of cancer: integrating quantitative models. Nat Rev Cancer. (2015) 15(12):730–45. doi: 10.1038/nrc4029 26597528

[22] Gatenby RA Maini PK. Mathematical oncology: cancer summed up. Nature (2003) 421(6921):321. doi: 10.1038/421321a 12540881

[23] Rockne RC Hawkins-Daarud A Swanson KR Sluka JP Glazier JA Macklin P. The 2019 mathematical oncology roadmap. Phys Biol (2019) 16(4):41005. doi: 10.1088/1478-3975/ab1a09 PMC6655440 30991381

[24] Aherne NJ Dhawan A Scott JG Enderling H. Mathematical oncology and it’s application in non melanoma skin cancer–A primer for radiation oncology professionals. Oral Oncol (2020) 103:104473. doi: 10.1016/j.oraloncology.2019.104473 32109841

[25] Alfonso JCL Parsai S Joshi N Godley A Shah C Koyfman SA. Temporally feathered intensity-modulated radiation therapy: A planning technique to reduce normal tissue toxicity. Med Phys (2018) 45(7):3466–74. doi: 10.1002/mp.12988 PMC6041138 29786861

[26] Leder K Pitter K LaPlant Q Hambardzumyan D Ross BD Chan TA. Mathematical modeling of PDGF-driven glioblastoma reveals optimized radiation dosing schedules. Cell (2014) 156(3):603–16. doi: 10.1016/j.cell.2013.12.029 PMC3923371 24485463

[27] Dean JA Tanguturi SK Cagney D Shin KY Youssef G Aizer A. Phase I study of a novel glioblastoma radiation therapy schedule exploiting cell-state plasticity. Neuro Oncol (2022) 25 (6):1100–12. doi: 10.1093/neuonc/noac253/6834115 PMC10237407 36402744

[28] Prokopiou S Moros EG Poleszczuk J Caudell J Torres-Roca JF Latifi K. A proliferation saturation index to predict radiation response and personalize radiotherapy fractionation. Radiat Oncol (2015) 10(1):1–8. doi: 10.1186/s13014-015-0465-x 26227259 PMC4521490

[29] Poleszczuk J Walker R Moros EG Latifi K Caudell JJ Enderling H. Predicting patient-specific radiotherapy protocols based on mathematical model choice for proliferation saturation index. Bull Math Biol (2018) 80(5):1195–206. doi: 10.1007/s11538-017-0279-0 28681150

[30] Sunassee ED Tan D Ji N Brady R Moros EG Caudell JJ. Proliferation Saturation Index in an adaptive Bayesian approach to predict patient-specific radiotherapy responses. Int J Radiat Biol (2019) 95(10):1421–6. doi: 10.1080/09553002.2019.1589013 PMC7081883 30831050

[31] Zahid MU Mohamed ASR Latifi K Rishi A Harrison LB Fuller CD. Proliferation saturation index to characterize response to radiation therapy and evaluate altered fractionation in head and neck cancer. Appl Radiat Oncol (2021) 10(1):32–9. doi: 10.37549/ARO1272

[32] Enderling H Alfonso JCL Moros E Caudell JJ Harrison LB. Integrating mathematical modeling into the roadmap for personalized adaptive radiation therapy. Trends Cancer (2019) 5(8):467–4. doi: 10.1016/j.trecan.2019.06.006 31421904

[33] Brady R Enderling H. Mathematical models of cancer: when to predict novel therapies, and when not to. Bull Math Biol (2019) 81(10):3722–31. doi: 10.1007/s11538-019-00640-x PMC6764933 31338741

[34] Zahid MU Mohamed AS R Caudell JJ Harrison LB Fuller CD Moros EG. Dynamics-adapted radiotherapy dose (DARD) for head and neck cancer radiotherapy dose personalization. J Personalized Med (2021) 11(11):1124. doi: 10.3390/jpm11111124 PMC8622616 34834476

[35] Zahid MU Mohsin N Mohamed ASRR Caudell JJ Harrison LB Fuller CD. Forecasting individual patient response to radiotherapy in head and neck cancer with a dynamic carrying capacity model. Int J Radiat Oncol Biol Phys (2021) 111(3):693–704. doi: 10.1016/j.ijrobp.2021.05.132 PMC8463501 34102299

[36] Araujo RP McElwain DLS. A history of the study of solid tumour growth: the contribution of mathematical modelling. Bull Math Biol (2004) 66(5):1039–91. doi: 10.1016/j.bulm.2003.11.002 15294418

[37] Brú A Albertos S Subiza JL García-Asenjo JL Brú I. The universal dynamics of tumor growth. Biophys J (2003) 85(5):2948–61. doi: 10.1016/S0006-3495(03)74715-8 PMC1303573 14581197

[38] Guiot C Degiorgis PG Delsanto PP Gabriele P Deisboeck TS. Does tumor growth follow a “universal law”? J Theor Biol (2003) 225(2):147–51. doi: 10.1016/S0022-5193(03)00221-2 14575649

[39] Guiot C Delsanto PP Carpinteri A Pugno N Mansury Y Deisboeck TS. The dynamic evolution of the power exponent in a universal growth model of tumors. J Theor Biol (2006) 240(3):459–63. doi: 10.1016/j.jtbi.2005.10.006 16324717

[40] Gerlee P. The model muddle: in search of tumor growth laws. Cancer Res (2013) 73(8):2407–11. doi: 10.1158/0008-5472.CAN-12-4355 23393201

[41] Benzekry S Tracz A Mastri M Corbelli R Barbolosi D Ebos JML. Modeling spontaneous metastasis following surgery: An in *vivo*-in silico approach. Cancer Res (2016) 76(3):535–47. doi: 10.1158/0008-5472.CAN-15-1389 PMC5846333 26511632

[42] Laleh NG Loeffler CML Grajek J Staňková K Pearson AT Muti HS. Classical mathematical models for prediction of response to chemotherapy and immunotherapy. PloS Comput Biol (2022) 18(2):e1009822. doi: 10.1371/journal.pcbi.1009822 35120124 PMC8903251

[43] McMahon SJ. The linear quadratic model: usage, interpretation and challenges. Phys Med Biol (2018) 64(1):01TR01. doi: 10.1088/1361-6560/aaf26a 30523903

[44] van Leeuwen CM Oei AL Crezee J Bel A Franken NAP Stalpers LJA. The alfa and beta of tumours: a review of parameters of the linear-quadratic model, derived from clinical radiotherapy studies. Radiat Oncol (2018) 13(1):1–11. doi: 10.1186/s13014-018-1040-z 29769103 PMC5956964

[45] Tozer GM Myers R Cunningham VJ. Radiation-induced modification of blood flow distribution in a rat fibrosarcoma. Int J Radiat Biol (1991) 60(1–2):327–34. doi: 10.1080/09553009114552081 1677989

[46] Friedman EJ. Immune modulation by ionizing radiation and its implications for cancer immunotherapy. Curr Pharm Des (2002) 8(19):1765–80. doi: 10.2174/1381612023394089 12171547

[47] Byun DJ Tam MM Jacobson AS Persky MS Tran TT Givi B. Prognostic potential of mid-treatment nodal response in oropharyngeal squamous cell carcinoma. Head Neck. (2020) 43(1):173–81. doi: 10.1002/hed.26467 PMC9879731 32964574

[48] Eschrich SA Pramana J Zhang H Zhao H Boulware D Lee JH. A gene expression model of intrinsic tumor radiosensitivity: prediction of response and prognosis after chemoradiation. Int J Radiat Oncol Biol Phys (2009) 75(2):489–96. doi: 10.1016/j.ijrobp.2009.06.014 PMC3038688 19735873

[49] Wallgren A. Late effects of radiotherapy in the treatment of breast cancer. Acta Oncologica (1992) 31(2):237–42. doi: 10.3109/02841869209088909 1622640

[50] Coia LR Myerson RJ Tepper JE. Late effects of radiation therapy on the gastrointestinal tract. Int J Radiat Oncol Biol Phys (1995) 31(5):1213–36. doi: 10.1016/0360-3016(94)00419-L 7713784

[51] Cooper JS Fu K Marks J Silverman S. Late effects of radiation therapy in the head and neck region. Int J Radiat Oncol Biol Phys (1995) 31(5):1141–64. doi: 10.1016/0360-3016(94)00421-G 7713779

[52] Gerlee P Altrock PM Malik A Krona C Nelander S. Autocrine signaling can explain the emergence of Allee effects in cancer cell populations. PloS Comput Biol (2022) 18(3):e1009844. doi: 10.1371/journal.pcbi.1009844 35239640 PMC8923455

[53] Kimmel GJ Locke FL Altrock PM. The roles of T cell competition and stochastic extinction events in chimeric antigen receptor T cell therapy. Proc R Soc B (2021) 288(1947). doi: 10.1098/rspb.2021.0229 PMC8059581 33757357

[54] Liney GP Whelan B Oborn B Barton M Keall P. MRI-linear accelerator radiotherapy systems. Clin Oncol (2018) 30(11):686–91. doi: 10.1016/j.clon.2018.08.003 30195605

[55] Hall WA Small C Paulson E Koay EJ Crane C Intven M. Magnetic resonance guided radiation therapy for pancreatic adenocarcinoma, advantages, challenges, current approaches, and future directions. Front Oncol (2021) 11:628155. doi: 10.3389/fonc.2021.628155 34046339 PMC8144850

[56] Lv J Chen Y Zhou G Qi Z Tan KRL Wang H. Liquid biopsy tracking during sequential chemo-radiotherapy identifies distinct prognostic phenotypes in nasopharyngeal carcinoma. Nat Commun (2019) 10(1):1–10. doi: 10.1038/s41467-019-11853-y 31477699 PMC6718666

[57] Chaudhuri AA Binkley MS Osmundson EC Alizadeh AA Diehn M. Predicting radiotherapy responses and treatment outcomes through analysis of circulating tumor DNA. Semin Radiat Oncol (2015) 25(4):305–12. doi: 10.1016/j.semradonc.2015.05.001 PMC4575502 26384278

[58] Earland N Chen K Semenkovich NP Chauhan PS Zevallos JP Chaudhuri AA. Emerging roles of circulating tumor DNA for increased precision and personalization in radiation oncology. Semin Radiat Oncol (2023) 33(3):262–78. doi: 10.1016/j.semradonc.2023.03.004 PMC12412029 37331781

[59] Gneiting T Raftery AE. Weather forecasting with ensemble methods. Sci (1979) (2005) 310(5746):248–9. doi: 10.1126/science.1115255 16224011

[60] Leutbecher M Palmer TN. Ensemble forecasting. J Comput Phys (2008) 227(7):3515–39. doi: 10.1016/j.jcp.2007.02.014

[61] Wu H Levinson D. The ensemble approach to forecasting: A review and synthesis. Transp Res Part C Emerg Technol (2021) 132:103357. doi: 10.1016/j.trc.2021.103357

[62] Araújo MB New M. Ensemble forecasting of species distributions. Trends Ecol Evol (2007) 22(1):42–7. doi: 10.1016/j.tree.2006.09.010 17011070

[63] Albuquerque PHM Peng Y da Silva JPF. Making the whole greater than the sum of its parts: A literature review of ensemble methods for financial time series forecasting. J Forecast (2022) 41(8):1701–24. doi: 10.1002/for.2894

