# Supplementary material for: Mathematical modeling of radiotherapy: impact of model selection on estimating minimum radiation dose for tumor control
Source: Front Oncol. 2023 Oct 9;13:1130966. doi: 10.3389/fonc.2023.1130966 (PMC10600389; doi:10.3389/fonc.2023.1130966)
Supplement: Supplementary file 1 [file DataSheet_1.pdf]

Supplementary Information
Mathematical modeling of radiotherapy: Impact of model selection on estimating minimum radiation dose for tumor control

Achyudhan R. Kutuva[1], Jimmy Caudell[2], Kosj Yamoah[2], Heiko Enderling[1,2], Mohammad U. Zahid[1]

[1]Quantiative Personalized Oncology Lab, H. Lee Moffitt Cancer Center & Research Institute, Department of Integrated Mathematical Oncology, Tampa, FL, USA; [2]H. Lee Moffitt Cancer Center & Research Institute, Department of Radiation Oncology, Tampa, FL, USA

Supplementary Methods

Combined DVR-CCR Model

We attempted to combine the DVR and CCR models into a single model where the effect of each RT fraction is modeled as an instantaneous reduction in tumor volume and tumor carrying capacity, as modeled in the independent DVR and CCR models.

We analyzed the feasibility of using this model by searching for regions of the (λ, α, δ) parameter space where V/K remained ≤ 1. This was done by sampling from $\alpha \in (0,0.4)$ Gy^{-1} with a step size of 0.01 Gy^{-1}, $\lambda \in (0, 0.3)$ day^{-1} with a step size of 0.01 day^{-1}, and $\delta \in (0,0.2)$ with step size of 0.01. This means that a total of 24,000 parameter sets were analyzed.

Supplementary Figures

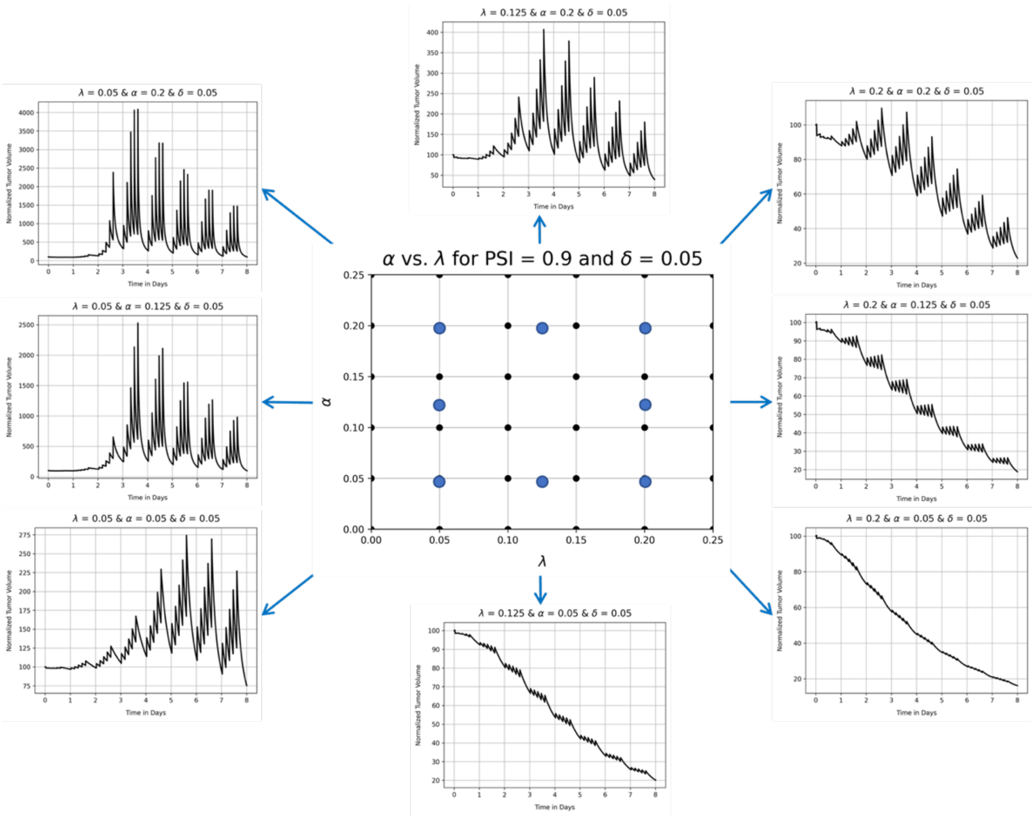

SI Figure 1. Combined DVR-CCR model simulations. Representative tumor volume trajectories from the combined DVR-CCR model for PSI = 0.9 and $\delta = 0.05$ across 8 (λ,α) pairs. In 6 out of the 8 pairs we observe unrealistic spikes and large oscillations in tumor volume when the next RT dose is simulated.

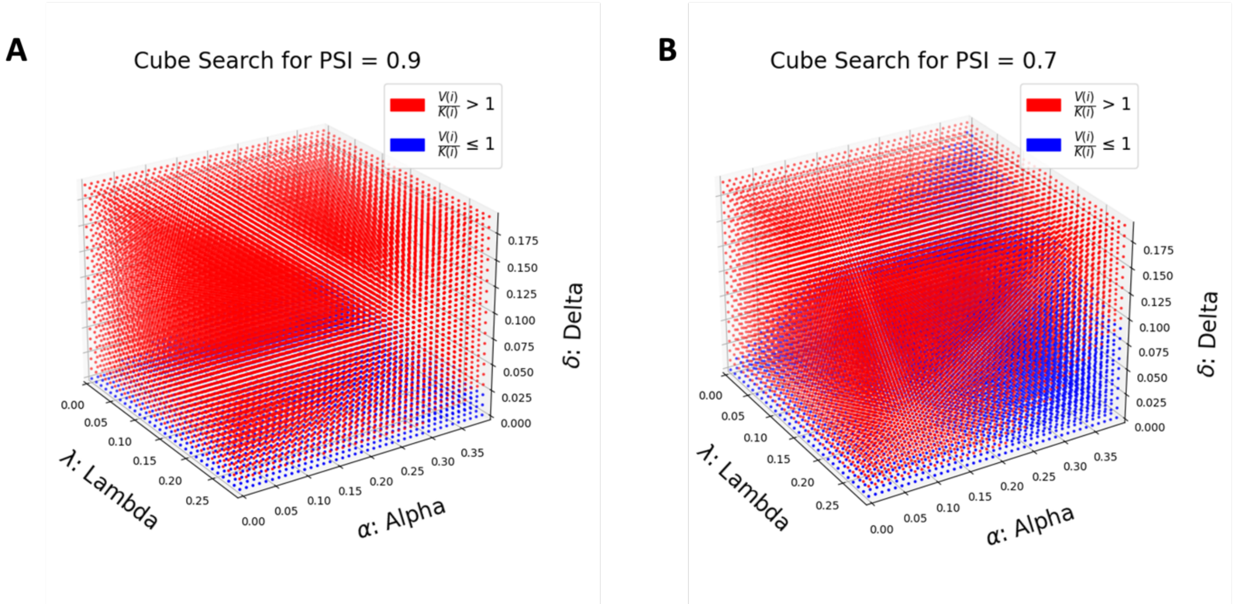

SI Figure 2. Cube search for combined DVR-CCR model. A Results of cube search across λ, α, and δ with PSI = 0.9 where red dots indicate simulations where V/K > 1 at some point during the simulation (resulting in unrealistic tumor volume dynamics) and blue dots indicate simulations where V/K ≤ 1 throughout the simulation. 86.9% of simulations yielded unrealistic dynamics. **B** Results of cube search across λ, α, and δ with PSI = 0.7 where red dots indicate simulations where V/K > 1 at some point during the simulation (resulting in unrealistic tumor volume dynamics) and blue dots indicate simulations where V/K ≤ 1 throughout the simulation. 65.1% of simulations yielded unrealistic dynamics.

Supplementary Table 1. Fitted coefficients for DVR model with form $D_{min} = \mathrm{a} * \mathrm{e}^{\mathrm{b}*\alpha} + \mathrm{c}$

PSI	a	b	c
**0.7**	588.35	-49.49	9.14
**0.8**	866.02	-49.81	12.55
**0.9**	1200.45	-47.67	19.07

Supplementary Table 2. Fitted coefficients for DVR model with form $D_{min} = a * e^{b*PSI} + c$

α	a	b	c
**0.08**	0.01	9.64	15.64
**0.1**	0.00	12.17	12.18
**0.12**	0.00	14.43	10.33

Supplementary Table 3. Fitted coefficients for CCR model with form $D_{min} = \mathrm{a} * \mathrm{e}^{\mathrm{b}*\delta} + \mathrm{c}$

PSI	**a**	**b**	**c**
**0.7**	142.1	-41.5	25.4
**0.8**	131.8	-44.8	23.6
**0.9**	113.6	-45.1	20.8

Supplementary Table 4. Fitted coefficients for CCR model with form $D_{min} = a * e^{b*PSI} + c$

**δ**	**a**	**b**	**c**
**0.03**	0.01	9.64	15.64
**0.05**	0.00	12.17	12.18
**0.07**	0.00	14.43	10.33
